# Supplementary material for: Crosslinked Pore-Filling Anion Exchange Membrane Using the Cylindrical Centrifugal Force for Anion Exchange Membrane Fuel Cell System
Source: Polymers (Basel). 2020 Nov 23;12(11):2758. doi: 10.3390/polym12112758 (PMC7700159; doi:10.3390/polym12112758)
Supplement: Supplementary file 1 [file polymers-12-02758-s001.pdf]

Supporting Information

Crosslinked Pore-filling Anion Exchange Membrane using the Cylindrical Centrifugal Force for Anion Exchange Membrane Fuel Cell System

Tae Yang Son[1], Tae Hyun Kim[2] and Sang Yong Nam[1,*]

[1]Department of Materials Engineering and Convergence Technology, Engineering Research Institute, Gyeongsang National University, Jinju, Republic of Korea; kr6620@naver.com (T.Y.S.)

[2]Oragnic Material Synthesis Laboratory, Department of Chemistry, Incheon National University, Incheon, Republic of Korea; tkim@inu.ac.kr (T.H.K.)

[*]Correspondence: walden@gnu.ac.kr (S.Y.N); Tel.: +82-55-772-1657; Fax: +82-55-772-1659

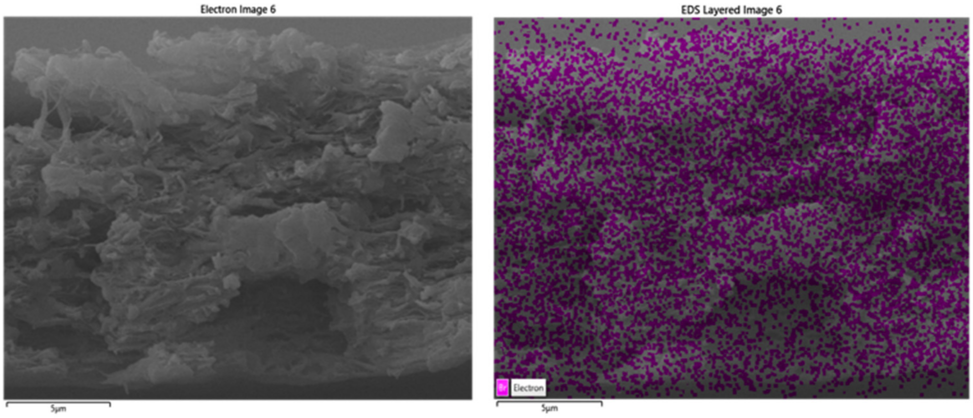

(a) (b)

Figure S1. FE-SEM and EDS images of crosslinked pore-filled membrane (cross-section): (a) FE-SEM image of PF-XAc-PPO70_25 and (b) EDS image of PF-XAc-PPO70_25

As can be seen in Figure S1, it was confirmed that Br ions present in the prepared crosslinked pore-filling membrane were distributed throughout the cross-section. It can be seen that the anion exchange polymer is well impregnated into the porous support.
